# Calculating Set-Volume for the Limb Muscles with the Performance of Multi-Joint Exercises: Implications for Resistance Training Prescription

**DOI:** 10.3390/sports7070177

**Published:** 2019-07-22

**Authors:** Brad J. Schoenfeld, Jozo Grgic, Cody Haun, Takahiro Itagaki, Eric R. Helms

**Affiliations:** 1 Health Sciences Department, City University of New York, Lehman College, Bronx, NY 10468, USA; 2 Institute for Health and Sport (IHES), Victoria University, Melbourne 3011, Australia; 3 Department of Exercise Science, LaGrange College, LaGrange, GA 30240, USA; 4 School of Sport and Recreation, Sport Performance Research Institute New Zealand, Auckland University of Technology, Auckland 1010, New Zealand

**Keywords:** muscle development, exercise prescription, electromyography

## Abstract

Resistance training volume, determined by the number of sets performed (set-volume) is considered one of the key variables in promoting muscle hypertrophy. To better guide resistance exercise prescription for weekly per-muscle training volume, the purpose of this paper is to provide evidence-based considerations for set-volume ratios between multi-joint (MJ) and single-joint (SJ) exercises so that practitioners can better manage prescription of training volume in program design. We analyzed this topic from three primary areas of focus: (1) biomechanical and physiological factors; (2) acute research; and (3) longitudinal research. From a biomechanical and physiological standpoint, when considering force production of different muscle groups, the moment arm of a given muscle, “motor abundance”, the link between biomechanics and exercise-induced fatigue, as well as the amount of time in voluntary muscle activation, a logical rationale can be made for SJ exercises producing greater hypertrophy of the limb muscles than MJ exercises (at least from specific exercises and under certain conditions). This would mean that sets for a MJ exercise should be counted fractionally for select muscles compared to an SJ exercise (i.e., less than a 1:1 ratio) when prescribing set-volumes for given muscles. When considering results from acute studies that measured muscle activation during the performance of SJ and MJ exercises, it seems that MJ exercises are not sufficient to maximize muscle activation of specific muscles. For example, during performance of the leg press and squat, muscle activation of the hamstrings is markedly lower than that of the quadriceps. These results suggest that a 1:1 ratio cannot be assumed. Current longitudinal research comparing the effects of training with MJ vs. SJ or MJ + SJ exercises is limited to the elbow flexors and the evidence is somewhat conflicting. Until more research is conducted to derive stronger conclusions on the topic, we propose the best advice would be to view set-volume prescription on a 1:1 basis, and then use logical rationale and personal expertise to make determinations on program design. Future research should focus on investigating longitudinal hypertrophic changes between MJ and SJ in a variety of populations, particularly resistance-trained individuals, while using site-specific measures of muscle growth to more systematically and precisely compute effective individualized set-volumes.

## 1. Introduction

During exercise, muscles produce forces that act on bony levers to carry out given movement patterns. Basic applied kinesiology classifies muscles as either prime movers, synergists, stabilizers, or antagonists. The following operational definitions can be used to describe these terms: A prime mover is involved in carrying out an action; a synergist contracts simultaneously with the prime mover to facilitate movement; an antagonist opposes the action of the prime mover(s), and; a stabilizer acts isometrically to provide structural support so that the movement can be performed efficiently.

Resistance exercises can be broadly classified as either multi-joint (MJ) or single joint (SJ). As the name implies, MJ exercises involve the use of more than one joint during performance; examples include squats, lunges, rows, and presses (e.g., the hip, knee, and ankle joints are all involved when performing the back squat). Alternatively, SJ exercises involve only one joint during performance; examples include biceps curls, triceps pushdowns, and leg extensions. A purported benefit of SJ exercises is that they afford the ability to better target an individual muscle compared to MJ exercises, thereby enhancing the hypertrophic stimulus for that muscle (or even a portion of the muscle) [1].

A recent meta-analysis endeavored to quantify the optimal number of sets per muscle per week (i.e., set-volume) for maximizing muscle hypertrophy [2]. In making their recommendations, muscle groups deemed prime movers during MJ and SJ were classified the same from a set standpoint. For example, a set of lat pulldowns (a MJ exercise) and a set of biceps curls (a SJ exercise) were both counted as 1 set (i.e., 1:1 ratio) when examining biceps brachii hypertrophy. Similarly, a set of squats was considered equal to a set of leg extensions when assessing quadriceps hypertrophy. Such an approach is consistent with a recent review that concluded equivalent hypertrophy can be achieved with the performance of MJ as compared to SJ exercise [3]. However, the veracity of this conclusion was subsequently challenged based on perceived misinterpretations and overextrapolations of supporting evidence [4].

Thus, while it appears clear that substantial hypertrophy of the limbs can be achieved by only performing MJ exercises, it remains equivocal whether additional benefits can be derived from SJ movements. Furthermore, it remains unclear whether both MJ and SJ exercises should be counted equally or differentially when providing recommendations for set-volume per muscle. This is a relevant metric to establish given that set-volume seems to be one of the most important training variables in resistance exercise prescription, both for muscle hypertrophy and health-related outcomes [5,6]. To better guide resistance exercise prescription for weekly per-muscle training volume, the purpose of this paper is to provide evidence-based considerations for set-volume ratios between MJ and SJ exercise of the upper and lower limb muscles. We attempted to draw conclusions by triangulating evidence from the following three primary areas of focus: biomechanical and physiological factors, acute research, and longitudinal research.

## 2. Biomechanical and Physiological Considerations

Biomechanical factors clearly influence the contribution of individual muscles to the total work performed during MJ exercises. The extent of their contribution will be dictated, at least in part, by the length-tension relationship, which states that a muscle’s capacity to produce force is predicated on the length at which it is held. Maximum force is often purported to occur at approximately resting length—the point where the maximum overlap of actin and myosin filaments occurs, which in turn facilitates the ability for optimal cross-bridge formation. However, this view neglects the complexity of in vivo kinetics, and thus may misrepresent the actual forces that occur throughout a range of movement during exercise performance. From a practical standpoint, the functional force-length range is predicated on myriad factors that include the absolute muscle length, the number of sarcomeres, tendon length and stiffness, the length of the moment arm, and the range of motion of the acting joint(s) [7]. When taking these factors into account, it becomes difficult to tease out how much a given muscle contributes to force production at a given point during performance of MJ exercise.

Moreover, the length-tension relationship includes both active forces from the myofilaments and passive forces from elastic components (such as titin, fascia, and tendon). It remains unclear how changes in active and passive muscular forces throughout a joint’s range of motion affect the hypertrophic response to resistance training. Given that multiple muscles are acting across a joint during MJ exercises, and given that these muscles may be functioning at different sarcomere lengths, it is conceivable that one or more muscles may be stretched or shortened to a length where active forces are minimal to nonexistent [7]. This would potentially impact the extent of hypertrophic stimulation of individual muscles, although the ramifications as to the effects on muscle development are yet to be elucidated.

Further confounding matters, there is evidence that stretching sarcomeres beyond their resting length can potentially enhance force output during exercise. When myofibers are stretched, the myofilaments (actin and myosin) are brought closer together, which in turn increases calcium sensitivity and henceforth cross-bridge attachment [8,9]. Thus, beneficial effects on force output can actually be seen at ~125–140% of resting length, as the greater probability of cross-bridge attachment from the proximity of myofilaments overcomes the decreased probability resulting from fewer myosin heads in the region of overlap [7]. Employing an in vitro model whereby the bullfrog plantaris muscle was subjected to a variety of maximal and submaximal fixed-end tetanic contractions, Holt and Azizi [10] reported a shift of the optimal length curve to longer lengths at low activation levels. These results were attributed to the varying effects of internal muscle mechanics, again demonstrating the inherent issues when attempting to determine the contribution of individual muscles during MJ exercise. Importantly, SJ exercise often can be altered to better allow training at a given muscle length, whereas during MJ exercise the lengths will necessarily change through the range of motion; this may afford an enhanced ability for SJ exercises to elicit greater hypertrophy in a given muscle. The implications are particularly relevant in biarticular muscles such as the hamstrings, rectus femoris, biceps brachii, and long head of the triceps, as rotation at one joint tends to shorten the muscle while rotation at the other joint tends to lengthen the same muscle [11].

Another important biomechanical consideration is the moment arm of a given muscle. While absolute length change affects muscle function during dynamic movement, the relative length change is of greatest consequence. The phenomenon can be best expressed by the fiber length-to-moment arm ratio, which represents a muscle’s sensitivity to joint rotation [11]. This property can vary extensively between muscles [12]. Whether such differences are magnified during MJ versus SJ remains unknown. However, given that muscles have different moment arms about different joints, the movement of each joint will differentially affect each muscle’s ability to produce force—both in terms of length and velocity—thereby providing a theoretical rationale by which muscle development may be differentially impacted by performance of an MJ versus SJ exercise.

Issues with “motor abundance”, operationally defined as the body’s attempt to find a unique solution to carry out a complex motor task [13], also must be taken into account during dynamic exercise performance. The nervous system controls gross movement patterns by a coordinated innervation of groups of muscles as opposed to innervating individual muscles on an isolated basis; differences in the number of moving joints in combination with the degrees of freedom thereby will cause proportional changes in all of the active muscles [14]. Accordingly, the body’s inherent drive to perform tasks as economically as possible will necessarily alter innervation patterns between MJ and SJ exercise; how these strategies ultimately affect muscular adaptations is yet to be determined.

The interplay between biomechanics and exercise-induced fatigue also needs to be considered from a hypertrophy standpoint. Muscle growth requires not only recruitment of a fiber, but also sufficient stimulation (i.e., fatigue) to induce a hypertrophic response. It is generally accepted that a minimum fatigue threshold for a given fiber must be reached to provide such a stimulus. There is some evidence that training at long muscle lengths promotes greater fatigue compared to training at a short length [7]. This may indicate that some limb muscles may not be adequately activated during MJ exercise due to their limited ability to work at longer lengths. However, other evidence suggests that fatigue may be fairly uniform across a spectrum of muscle lengths [15], calling into question whether this factor would have a meaningful effect on the adaptive response to MJ exercise.

Furthermore, individual anthropometrics and muscle moment arms can influence the relative demands of a muscle during an exercise [16]. This could differentially affect hypertrophic outcomes between individuals. In other words, an exercise could be more or less hypertrophic for a given muscle in one individual compared to another. Practically, however, when considering the similarity in human anatomy and physiology, weekly muscle set-volume could still be counted in the same manner since overall muscle excursion and joint actions would be mostly the same qualitatively, even though the relative difficulty might be different. For example, complete voluntary flexion of the elbow with a 20 kg load in the hand requires biceps brachii contraction and this joint action can be counted as a set for this muscle. Yet, due to a shorter or longer biceps muscle moment arm, an individual may have to produce more or less biceps force to complete the joint action against an applied resistance. Thus, a single set of repetitions of an exercise could still be counted as such toward weekly set-volume for a prime mover, although it is important to appreciate that the relative difficulty of a single set may be different for different individuals.

Finally, relevant physiological factors warrant mention here. Mechanical tension produced and experienced by a muscle fiber is the key hypertrophic stimulus incurred through resistance exercise [17]. Tension can be either actively produced or passively experienced by a muscle fiber. Both types of tension seem necessary for maximum hypertrophy [18]. As individual sarcomeres shorten within a myofibril, pulling forces are generated via actin-myosin crossbridge formation. These forces are transduced laterally and longitudinally to muscle fascia via integrin proteins [19] and tensile forces are also experienced passively during resistance exercise. Passive tension or stretch under load has been shown to induce hypertrophy in animal models [20]. Thus, if a muscle maximally or near-maximally lengthens and shortens during an exercise, it seems prudent to characterize the exercise to count as a set toward that muscle or muscles. Active tension results from stretching forces that occur during different whole-muscle actions (i.e., concentric, isometric, eccentric).

Importantly, the amount of time voluntary activation of a muscle occurs during different whole-muscle actions directly impacts the gross tension experienced during a single set of repetitions of an exercise. For example, a 5 s eccentric muscle action, 2 s isometric muscle action, and 1 s concentric muscle action results in different gross tension experienced by fibers in a given muscle compared to 1 s of each action when the same joint range of motion is covered. Therefore, repetition tempo and total repetition duration can also influence the relative difficulty of a single set and the hypertrophic outcomes over time [21]. Notwithstanding, a muscle undergoing voluntary activation yet working isometrically or through a partial range of motion during an exercise dominated by other musculature is still experiencing tension. This warrants consideration when counting sets for various exercises as fractions of sets for different muscles that experience notable tension, but that are not prime movers in reference to the primary joint action. For example, the bench press exercise could count as a set for the pectoralis muscles but a fraction of a set toward the triceps and front deltoids. However, this likely depends on the individual and the technical execution of the exercise. Thus, this presents a difficult challenge to objectively quantify in the practical setting. Although these factors can influence adaptive outcomes and the relative stress of a given set, it stands to reason that, from a practical standpoint, an exercise that involves a joint’s prime movers can logically be counted toward weekly set-volume and a fractional set-counting strategy could potentially be employed, as discussed later.

In summarizing the biomechanical and physiological considerations, a logical rationale can be made for SJ exercise producing greater hypertrophy of the limb muscles than MJ, at least in certain exercises and under certain conditions. If so, this would mean that sets for an MJ exercise should be counted fractionally for select muscles compared to an SJ exercise (i.e., less than a 1:1 ratio) when prescribing set-volumes for given muscles. However, the ability to draw conclusions from theoretical constructs of biomechanics and physiology requires a high degree of speculation from an applied exercise standpoint, and therefore must be viewed in the context of more direct evidence on the topic.

## 3. Acute Studies

Electromyography (EMG) is commonly used to assess myoelectric activity during exercise performance. EMG measures depolarization and hyperpolarization that occur across the sarcolemma [22]. In this way, EMG provides a gauge of neural drive into the muscle and, conceivably, provides insight into the contribution of a given muscle to the total work performed during exercise.

The compelling body of studies using EMG show that MJ exercises are not sufficient to maximize myoelectric activity of certain muscles in certain cases. For example, studies show that hamstrings EMG amplitude is significantly and markedly (~twofold) lower than that of the quadriceps during performance of the squat [23,24] and the leg press [25,26]. Moreover, EMG amplitudes have been found to be substantially greater when performing SJ exercise that directly targets the hamstrings (i.e., leg curl, stiff leg deadlift) compared to MJ lower-body exercise (i.e., squat, leg press) [27,28]. These data have a logical rationale from a functional anatomical perspective given that the hamstring muscles act as prime movers during both hip extension and knee flexion, which in turn suggests its length would remain relatively constant during MJ lower-body exercise [29]. Adding further insight to the findings, Mendiguchia et al. [30] found differences in region-specific activation within the proximal, middle, and distal regions of the hamstrings between the leg curl and the lunge as determined by magnetic resonance imaging (MRI), indicating that SJ and MJ may not be interchangeable when determining prescription for exercise volume for this muscle complex.

A number of studies have reported that EMG amplitude of the rectus femoris is significantly higher during performance of knee extension exercise compared to barbell squats and leg press exercises [28,31]. For example, Andersen et al. [28] reported normalized EMG amplitudes of 68% maximal voluntary contraction for the rectus femoris in the leg extension versus just 39% in the leg press and 27% in the squat. Similar findings have been shown using MRI, where contrast shifts (i.e., alterations in signal intensity) indicate preferential rectus femoris activation [32] as well as muscle damage [33] during knee extension exercise compared to MJ exercise. Moreover, Yamashita et al. [34] found that rectus femoris myoelectric activity was markedly depressed during combined hip and knee extension compared to the vastus medialis. In addition, there are studies showing that SJ exercise produces greater overall EMG myoelectric activity and MRI contrast shifts compared to MJ [28,32], although other studies contradict these findings [35,36]. Overall, these data suggest that simply performing MJ movements is not sufficient to maximally engage the rectus femoris and perhaps the quadriceps as a whole, which in turn potentially may have implications on long-term muscular development.

Several studies have reported ~twofold greater myoelectric activity of the pectoralis major compared to the triceps brachii during performance of the bench press [37,38]. Similarly, the lat pulldown and seated row result in greater myoelectric activity in the latissimus dorsi compared to the biceps brachii, although the disparity in EMG amplitude between muscles diminishes when a supinated grip is employed [39,40]. Interestingly, the narrowing of the latissimus dorsi to biceps brachii ratio in EMG amplitude occurred primarily due to a reduction in myoelectric activity of the latissimus dorsi as opposed to an increase in biceps brachii activity. There is evidence that EMG amplitude in the arm muscles during MJ exercise can be increased by adopting an internal focus (i.e., focusing on the activated muscle rather than on the weight by using the ‘mind-muscle connection’) [41,42], and that this strategy may enhance muscle growth [43]. Thus, the performance strategy may play a role in the contribution of individual muscles during MJ exercise and, in turn, their subsequent hypertrophy.

Collectively, evidence from studies using EMG indicates that SJ exercises elicit greater myoelectric activity of various aspects of the limb musculature. Based on this interpretation—at least in the specific exercise examples provided—the number of sets for MJ cannot be considered equal to that from SJ exercise from a hypertrophy standpoint. Therefore, the ratio for a prescription would be less than 1:1. However, it is important to note that the extent to which hypertrophic potential can be inferred from EMG analysis remains speculative. Several studies have shown that muscle activation, as measured by transverse relaxation time (T2)-weighted MRI, correlates with subsequent muscle hypertrophy of the elbow extensors [44,45] and quadriceps femoris [46]. If we consider that the activation measured by T2 weighted MRI significantly correlates with EMG data [47], it is tempting to speculate that SJ exercises are superior for hypertrophy of the limb muscles. Thus, volume for these muscles in MJ exercises must be accounted for at a lower percentage on a set basis. However, the validity of MRI has been called in question as a measure for predicting long-term muscular adaptations [48], raising some skepticism as to the practical implications of its correlation with EMG.

Importantly, causality cannot necessarily be determined from correlative data, and alternative evidence casts doubt on the validity for using EMG analysis to predict hypertrophic outcomes. Specifically, low-load training consistently shows lower EMG amplitudes compared to high-load training [26,49], yet the compelling body of literature indicates that longitudinal hypertrophic changes are similar regardless of the magnitude of loading provided the sets are carried out to muscle failure [50]. This seemingly refutes the predictive ability of EMG for longitudinal outcomes and, at the very least, suggests that caution should be exercised when attempting to extrapolate findings to changes in hypertrophy. Moreover, EMG amplitudes are generally based on maximal isometric voluntary contractions for each individual muscle analyzed; how this relates to the forces produced during dynamic exercise remains questionable.

## 4. Longitudinal Studies

It can be argued that the best way to determine how to account for set-volume of a given muscle during MJ exercise is to examine the results of longitudinal research (i.e., training interventions) on the topic. Accordingly, several studies have compared hypertrophic changes involving MJ versus SJ exercise. Barbalho and colleagues carried out a series of 8-week experiments on the topic that involved a variety of different populations including untrained young men [51], untrained young women [52], young women with previous resistance training experience [53], and young men with previous resistance training experience [54]. Results of these studies were somewhat disparate. For example, the study in untrained young men [51] showed significantly greater increases in flexed arm circumference for the group performing a combination of SJ and MJ exercises compared to performing MJ movements alone (5.2% versus 4.0%, respectively; *p* = 0.001). Similar findings were seen in their study on untrained young women [52], with significantly greater increases in flexed arm circumference favoring SJ + MJ versus MJ (4.4% versus 3.5%, respectively; *p* = 0.002). Alternatively, resistance-trained women showed similar increases in flexed arm circumference regardless of whether subjects performed SJ + MJ versus just MJ (1.5% MJ and 1.6% MJ + SJ), suggesting that previous resistance training experience may negate any hypertrophic advantage of SJ exercises [53]. In support of this hypothesis, both Barbalho et al. [54] and De Franca et al. [55] reported similar increases in flexed arm circumference following 8-week RT programs in cohorts of resistance-trained men regardless of whether they performed MJ + SJ or MJ alone. It should be noted that the volume between conditions was not equated in the majority of these studies. Given the well-established dose-response relationship between volume and hypertrophy [2], this raises the possibility that greater training volumes may have unduly influenced results in the studies showing favorable effects for SJ exercises. Importantly, flexed arm circumference can be considered a relatively crude measure of hypertrophy, with limited ability to detect the subtle changes in muscle growth that would be expected in these relatively short-term protocols. Thus, findings should be interpreted with a degree of circumspection.

Recently, Bezerra et al. [56] randomized 30 untrained, older (age > 55 years) men and women to perform either MJ exercise (cable chest press and seated row), a combination of MJ + SJ exercises (cable chest press, seated row, biceps curl, and triceps extension) or a non-training control. The study employed a low volume protocol, with the MJ group performing 2 sets per exercise and MJ + SJ group performing 1 set per exercise with a 12-repetition maximum load. The training was conducted 3 times per week for 8 weeks. Changes in upper limb lean mass were measured by dual X-ray absorptiometry (DXA). Interestingly, neither group achieved any post-study increase in lean mass of the upper limbs. While DXA is generally considered as a viable modality to assess muscle mass and is clearly superior to flexed arm circumference in this regard, its ability to detect subtle hypertrophic changes is inferior compared to site-specific measures such as MRI, computerized tomography, and ultrasound [57,58]. Given the lack of appreciable increases in lean mass across the study period, it can be speculated that the training stimulus was suboptimal to induce hypertrophy, at least as determined by DXA; the findings, therefore, should be interpreted cautiously as to the practical implications for determining set-volume in SJ versus MJ exercise.

To date, three studies have investigated hypertrophic changes between MJ + SJ vs. MJ using a site-specific measure of hypertrophy. Gentil et al. [59] randomized 29 untrained young men to perform either MJ exercises consisting of the lat pulldown and the bench press, or a group that performed these same exercises as well as elbow flexion and elbow extension. Subjects performed 3 sets of 8–12 repetitions for each exercise. The training was conducted twice a week for 10 weeks. Muscle thickness of the biceps brachii was measured using B-mode ultrasonography. Results showed that both groups significantly increased muscle thickness to a similar extent (6.5% for MJ versus 7.0% for MJ + SJ). The same laboratory carried out a follow-up study, whereby 29 untrained young men were randomized to perform either a MJ exercise involving the elbow flexors (lat pulldown) or a SJ exercise involving the elbow flexor muscles (biceps curl) [60]. Subjects trained twice a week, performing 3 sets of 8–12 repetitions. As in the previous study, muscle thickness was evaluated via B-mode ultrasonography. After 10 weeks, both groups showed similar increases in muscle thickness (6.1% and 5.8% for MJ and SJ, respectively). Most recently, Mannarino et al. [61] carried out a within-subject design whereby the upper extremities of 10 untrained men were randomized to perform SJ arm curls in one limb and MJ dumbbell rows in the contralateral limb. The program consisted of 4–6 sets of 8–12 repetitions per exercise with sessions performed twice a week. After 8 weeks, muscle thickness of the elbow flexors was more than twofold greater in the arm that performed SJ compared to MJ exercise (11.1% versus 5.2%, respectively). Collectively, these conflicting findings preclude the ability to draw strong inferences on the topic. It should be noted that performance of the MJ exercise in these studies was carried out with a reverse grip which, as compared to a neutral grip, increases activation of the biceps brachii. It is conceivable that employing a pronated or neutral grip may alter results. Moreover, these findings are specific to the elbow flexors and cannot necessarily be extrapolated to other muscle groups such as those of the lower-body.

Although no study has directly compared hypertrophy of the hamstrings pursuant to SJ vs. MJ, there is some evidence that muscular development in this muscle complex is suboptimal from performance of MJ alone. Weiss et al. [62] randomized 40 untrained young men to perform 4 sets of barbell squats using either heavy, moderate, or light loading schemes. After the 7-week study period, significantly greater pre- to post-study changes in muscle thickness were seen for the quadriceps; however, no significant pre-to-post intervention increases were observed for the hamstrings. Bloomquist et al. [63] showed similar findings, reporting markedly greater increases in CSA of the quadriceps as compared to the hamstrings following 12 weeks of resistance training that included the half-squat or full-squat exercises. It cannot be determined whether the addition of an SJ exercise for the hamstrings may have elicited greater development of the muscle complex in either of these studies but, when combined with the aforementioned hamstrings EMG data, the results raise the prospect for a potential benefit.

A limitation of the current literature is that studies on the topic have all measured markers of muscle growth at a single site along the muscle, generally the midpoint. This is potentially problematic as there may be regional hypertrophic differences between SJ and MJ exercise that may not be accounted for in such measures. For example, Wakahara et al. [44] subjected 12 young men to a bench press protocol performed 3 days a week for 12 weeks. Post-study triceps growth, as measured by MRI at various sites along the posterior upper arm, was significantly greater in the mid-portion of the muscle compared to the proximal portion. The proximal portion corresponds to the long head of the triceps, which suggests this aspect did not receive sufficient stimulation due to length-tension factors during MJ exercise. Intriguingly, the same research group found greater hypertrophy of the proximal portion of the muscle (long head) following 12 weeks performance of the lying triceps extension, a SJ exercise, compared to the distal and mid-points [45]. Taking the findings of the two studies together, it is reasonable to conclude that SJ exercise produces greater hypertrophy in the long head of the triceps compared to MJ exercise alone.

The totality of longitudinal evidence suggests that some muscles (e.g., long head of the triceps, hamstrings) may derive somewhat greater hypertrophic benefits from SJ versus MJ, while in others (e.g., biceps brachii) the evidence is less clear. It is important to note that the extent of the longitudinal literature is very limited on the topic. Only two studies directly compared hypertrophy changes in MJ and SJ using site-specific measures of growth, and both focused on the biceps brachii. Thus, further research is needed to draw stronger inferences on the topic.

## 5. Additional Considerations

The time-course for muscle hypertrophy must also be considered when attempting to provide recommendations on set-volume for MJ exercise. Given the acute differences in muscle activation between SJ and MJ exercises (see the Acute Studies section), there remains a possibility that their performance also elicits different changes in muscle size over time. In one study, participants performed bench press, leg press, and biceps curl exercises in a training program that lasted for 20 weeks. Lean mass changes of the trunk, legs, and arms were measured using DXA pre, mid (10 weeks) and post-training [64]. At mid-point testing, only the lean mass of the arms increased from pre-training which may suggest that muscle hypertrophy of certain muscles may be more rapidly induced by performing SJ exercises. The caveat here is that the participants also performed the bench press exercise which produces large activation of the triceps muscle and therefore may have impacted these results. Moreover, there is a reduced motor learning curve for SJ compared to MJ exercise. Considering the untrained status of the participants, it, therefore, is possible that they were better able to induce greater peripheral overload and thus stimulate greater hypertrophy in the biceps brachii (trained with SJ exercise) versus the muscles of the trunk and legs (trained with MJ exercise).

In addition, it is conceivable that training status may have an effect on set-volume determinations for MJ exercise prescription. To date, longitudinal studies employing site-specific hypertrophy measures included only untrained individuals as study participants. Therefore, these results cannot necessarily be generalized to those with previous training experience. Anecdotally, bodybuilders commonly used both SJ and MJ exercises in their training routines. While these athletes base their training programs more on experimentation and intuition rather than on scientific evidence, emerging evidence indicates that various training practices employed by bodybuilders [65] also have considerable researched-based support [2,66]. Thus, it might be that the use of SJ exercises in a training program provides some additional benefits for muscle hypertrophy that have merely not yet been adequately explored in studies to date.

The range of motion (ROM) of an exercise also may be a factor when attempting to determine set-volume ratios for MJ versus SJ. Specifically, certain MJ exercises may involve a lesser excursion of the limbs than others. For example, a dumbbell bench press may allow for a larger elbow joint ROM compared to a barbell press; a narrow-grip row may incur a somewhat greater elbow flexion compared to a wide-grip row. Moreover, individual performance of a given exercise can also alter ROM about a given joint. For example, some individuals perform the squat with a more hip-dominant movement pattern, which in turn tends to reduce excursion at the knee joint. There is some evidence that training through a full ROM elicits a greater hypertrophic response compared to a partial ROM [63,67,68], although these findings are not universal [69]. The extent to which this variable may affect set-volume ratios is undetermined and warrants further investigation.

## 6. Conclusions

Based on the current literature, it would seem that hypertrophy of some limb muscles may be similar to the isolated performance of SJ or MJ movements, while for other muscles, additional hypertrophic benefits might be attained from SJ. Thus, set-volume prescription in this regard will be muscle-specific. For the biceps brachii, evidence on the topic is mixed with some studies showing equal hypertrophic effects between MJ and SJ exercise [59,60], and others showing a benefit from performing SJ exercise [61]. Importantly, it is not clear to what extent grip (supinated, pronated, or neutral) employed during MJ exercise involving the elbow flexors may influence results. For the triceps brachii, it can be speculated that the set-volume ratio of MJ:SJ may be a bit lower than for the biceps, particularly for the long head of the triceps. This notion is consistent with the combination of muscle biomechanics theory, EMG data [37,38], and longitudinal studies showing greater growth in the long head when using the lying triceps extension (i.e., SJ exercise) vs. the bench press (i.e., MJ exercise) [44,45]. The same extrapolations may apply to the quadriceps muscle given some of the EMG data showing higher amplitude during the performance of a SJ exercise, particularly in the rectus femoris. For the hamstrings, it would seem that the ratio would be the lowest given the biomechanical considerations and EMG findings, as well as the extrapolation of results from longitudinal training interventions [62]. To assist in practical application of the concepts discussed, Table 1 provides an example checklist to determine muscle emphasis in a given exercise.

When attempting to draw applied evidence-based recommendations from meta-analytic findings, practitioners should understand that even if it is more valid to count SJ and MJ exercises with different set ratios, doing so may be disadvantageous as the current meta-analytic data is based on relationships between volume and hypertrophy determined on a 1:1 basis. Until more research is conducted to derive stronger conclusions on the topic, we feel the best advice for practitioners is to continue to view set-volume prescription on a 1:1 basis, and then use logical rationale and personal expertise to make determinations on program design. Future research should focus on investigating longitudinal hypertrophic changes between MJ and SJ in a variety of populations, particularly resistance-trained individuals, using site-specific measures of muscle growth. Moreover, attempts should be made to establish reference standards for exercises that are deemed to maximally activate a given muscle throughout its ROM. Other exercises then can be assessed to determine their relative activation to the reference standard for a given muscle group, and hence assigned a fractional set-count for that muscle group on more of an individual basis.

## Figures and Tables

**Table 1:** Muscle emphasis checklist example.

Unique Exercise Name	Primay Joint Action	Majority of Rom Covered?	Primary Muscle	Other Joint Action	Majority of Rom Covered?	Primary Muscle
BACK SQUAT	KNEE EXTENSION	YES	QUADRICEPS	HIP EXTENSION	YES	GLUTEALS

**Legend**: This checklist can assist in classifying an exercise to emphasize a certain muscle based on the joint actions involved. The barbell squat is used as example to show application to a specific exercise.
